# Use of linkage to improve the completeness of the SIM and SINASC in the Brazilian capitals

**DOI:** 10.11606/S1518-8787.2017051000431

**Published:** 2017-11-16

**Authors:** Lívia Teixeira de Souza Maia, Wayner Vieira de Souza, Antonio da Cruz Gouveia Mendes, Aline Galdino Soares da Silva

**Affiliations:** I Universidade Federal de Pernambuco. Centro Acadêmico de Vitória. Núcleo de Saúde Coletiva. Recife, PE, Brasil; II Fundação Oswaldo Cruz. Instituto Aggeu Magalhães. Departamento de Saúde Coletiva. Recife, PE, Brasil

**Keywords:** Infant Mortality, Birth Registration, Mortality Registries, Systems Integration, Health Information Systems, Vital Statistics, Mortalidade Infantil, Registro de Nascimento, Registros de Mortalidade, Integração de Sistemas, Sistemas de Informação em Saúde, Estatísticas Vitais

## Abstract

**OBJECTIVE:**

To analyze the contribution of linkage between databases of live births and infant mortality to improve the completeness of the variables common to the Mortality Information System (SIM) and the Live Birth Information System (SINASC) in Brazilian capitals in 2012.

**METHODS:**

We studied 9,001 deaths of children under one year registered in the SIM in 2012 and 1,424,691 live births present in the SINASC in 2011 and 2012. The databases were related with linkage in two steps – deterministic and probabilistic. We calculated the percentage of incompleteness of the variables common to the SIM and SINASC before and after using the technique.

**RESULTS:**

We could relate 90.8% of the deaths to their respective declarations of live birth, most of them paired deterministically. We found a higher percentage of pairs in Porto Alegre, Curitiba, and Campo Grande. In the capitals of the North region, the average of pairs was 84.2%; in the South region, this result reached 97.9%. The 11 variables common to the SIM and SINASC had 11,278 incomplete fields cumulatively, and we could recover 91.4% of the data after linkage. Before linkage, five variables presented excellent completeness in the SINASC in all Brazilian capitals, but only one variable had the same status in the SIM. After applying this technique, all 11 variables of the SINASC became excellent, while this occurred in seven variables of the SIM. The city of birth was significantly associated with the death component in the quality of the information.

**CONCLUSIONS:**

Despite advances in the coverage and quality of the SIM and SINASC, problems in the completeness of the variables can still be identified, especially in the SIM. In this perspective, linkage can be used to qualify important information for the analysis of infant mortality.

## Abstract (pt)

**OBJETIVO:**

Analisar a contribuição do *linkage* entre os bancos de dados de nascidos vivos e óbitos infantis para a melhoria da completude das variáveis comuns ao Sistema de Informações sobre Mortalidade (SIM) e ao Sistema de Informação sobre Nascidos Vivos (Sinasc) nas capitais brasileiras no ano de 2012.

**MÉTODOS:**

Foram estudados 9.001 óbitos de menores de um ano registrados no SIM no ano de 2012 e 1.424.691 nascidos vivos em 2011 e 2012 contidos no Sinasc. As bases de dados foram relacionadas por meio do *linkage* em duas etapas – determinística e probabilística. Calculou-se o percentual de incompletude das variáveis comuns ao SIM e ao Sinasc antes e após o emprego da técnica.

**RESULTADOS:**

Foi possível relacionar 90,8% dos óbitos à sua respectiva declaração de nascido vivo, a maioria pareada deterministicamente. Constatou-se maior percentual de pares em Porto Alegre, Curitiba e Campo Grande. Nas capitais do Norte, a média de pares foi de 84,2%; no Sul, esse resultado chegou a 97,9%. As 11 variáveis comuns ao SIM e ao Sinasc apresentavam 11.278 campos incompletos cumulativamente, sendo possível recuperar 91,4% das informações após o emprego do *linkage*. Cinco variáveis apresentavam completude excelente no Sinasc em todas as capitais brasileiras e apenas uma no SIM antes do relacionamento. Após a aplicação dessa técnica, todas as 11 variáveis do Sinasc passaram ao status de excelência, enquanto o mesmo ocorreu em sete no SIM. A capital de nascimento associou-se significativamente ao componente do óbito na qualidade da informação.

**CONCLUSÕES:**

Apesar dos avanços na cobertura e qualidade do SIM e do Sinasc, ainda são identificados problemas de completude das variáveis, em especial no SIM. Nessa perspectiva, o emprego do *linkage* apresenta-se como estratégia para qualificação de informações importantes para análise das mortes infantis.

## INTRODUCTION

Child mortality is an important indicator of maternal and child health, being considered as a sentinel event because of its avoidability with the provision of adequate living conditions and care quality and access for pregnant women, the delivery, and newborns^1^.

Despite contemporary advances, infant mortality persists as a public health problem in the world, especially in poorer countries and regions, which has led the United Nations to include the reduction of child mortality by two-thirds as one of the eight Millennium Development Goals^1^.

The monitoring and evaluation of the compliance with the millennium goal of reducing infant mortality implies the availability and adequacy of health information, which is still a challenge to be faced by the health sector in the Americas^2^. In Brazil, the Mortality Information System (SIM) and the Live Birth Information System (SINASC) have been developed by the Ministry of Health in view of the need to know the epidemiological situation of deaths and births in the country^3^.

By identifying limitations in the coverage and quality of the information produced by these systems^4^ ^,^ ^5^, the Ministry of Health made investments that resulted in a marked improvement in the SIM and SINASC in relation to both the coverage and quality of their data over the last two decades^3,6–8^. However, this coverage is heterogeneous in the country, with large variations among the states and some with low percentages, particularly those located in the North and Northeast regions^9^.

The analysis of the health situation and planning actions to reduce child mortality is provided by the availability of information of adequate quality. The access to reliable data allows the identification of the conditions of births and deaths and their determinants with greater validity^10^.

Several aspects, such as the completeness of variables, reliability, and consistency, need to be analyzed in the evaluation of the quality of vital statistics^11^. Underreporting and the presence of unknown or unfilled variables also compromise the reliability of the data and, consequently, the use of real data on infant mortality in Brazil^12^.

In this perspective, we highlight that the analysis of completeness is an important dimension of the evaluation of the quality of the information, exposing the lack of care and importance given to the filling by health professionals, the absence of data in medical records, and even the lack of knowledge on certain information by the woman’s or the child’s companions^13^. These deficiencies are also the result of the access to health services, diagnostic technologies, and the ability of the medical professional to recognize the dynamics of the events that participated in the causal chain of death, as well as their relationship with the production of reliable statistics^14^.

Several authors have used linkage as a strategy to improve the quality of information, since this procedure allows the recovery of incomplete or inconsistent records, thus improving the completeness and reliability of the information provided by the SINASC and SIM^15–17^.

In addition, linkage has low operational cost and is ease to manage, which facilitates the more qualified analysis of the health situation and the monitoring of the prevalence of risk factors and their magnitude in the population of live births, also facilitating the planning and evaluation of the maternal and child health^18^ ^,^ ^19^.

In this perspective, this study aimed to analyze the contribution of linkage between databases of live births and infant mortality to improve the completeness of the variables common to the SIM and the SINASC in Brazilian capitals in 2012.

## METHODS

This is an observational, cross-sectional study carried out in the 26 Brazilian capitals and the Federal District, in which we analyzed the information related to 9,001 deaths of children under one year registered in the SIM in 2012 and 1,424,691 live births present in the SINASC in 2011 and 2012. The data entry documents for these systems are the declaration of death (DD) and the declaration of live birth (DLB).

We used the linkage technique as a methodological tool, applying the deterministic and probabilistic methods.

The first step of the linkage (deterministic) was performed from the identification of the unifying variable common to both systems, the DLB number. For this purpose, we used one of the research and reference functions (PROCV) provided in the software Microsoft^®^ Office Excel 2007.

For unpaired records at this step, we used probabilistic linkage using automated routines (standardization, linkage, and combination of files), based on common fields present in both databases, in order to identify the probability of a pair of records belonging to the same individual.

We applied the probabilistic method using the multi-step strategy, associated with a manual review of the doubtful pairs, seeking to classify them as true pairs or non-pairs. The blocking fields used were the soundex of the mother’s first name, the soundex of the mother’s last name, the child’s gender, and the mother’s age. For comparison, we use the mother’s name and the child’s birth date. The variables used as a decision criterion during the manual inspection of pairs were the residence address, neighborhood of residence, complement of the residence address, and age of the mother and the date of death and year of birth of the child.

All probabilistic step processing was performed using the software Reclink III^®^ version 3.0.4.4005, a free program that allowed us to associate files based on the probabilistic linkage of records. At the end of this process, the files from the deterministic and probabilistic steps were unified, followed by the filling of the incomplete fields in the SIM and SINASC.

We selected the variables common to the two databases to analyze the percentage of completeness, namely: sex and race of the child, age of the mother, education level of the mother, occupation of the mother, number of children born alive, number of stillbirths, type of pregnancy, length of pregnancy, birth weight, and type of delivery.

We defined data as incomplete when fields were not filled in or when they were marked as unknown. For each variable common to the systems, we analyzed the filling before and after linkage, categorizing them according to the criteria of incompleteness proposed by Romero and Cunha^12^: excellent (< 5%), good (5% to 9.9%), regular (10% to 19.9%), poor (20% to 49.9%), and very poor (≥ 50%).

In addition to the completeness aspect of the variables, considering the assumption that the better the quality of the information the greater the chance of linkage success, we analyzed the association between the pairing of the SIM and SINASC, the capital of residence of the child, and the component of infant mortality. This last one expresses the subgroups of the age of death of children under one year, classified as neonatal (from zero to 27 days of life) or post-neonatal (from 28 days to one year of life).

To this end, we considered as cases the infant deaths not paired to their respective DLB and as controls those whose birth and death records were concatenated. We calculated odds ratios (OR) to verify the association between non-pairing (outcome) between systems and independent variables (capital and child death component). We evaluated the significance of the differences between the proportion of pairs and non-pairs from the linkage of the databases using the chi-square test, with statistical significance (p < 0.05).

This study used secondary data from the SIM and SINASC provided by the Ministry of Health by the technical opinion from the Department of Health Surveillance (SVS/MS), the term of assignment and use of databases, and the signing of the statement of responsibility by the researchers. This work is part of the research named “*Determinantes da mortalidade infantil nas capitais brasileiras: Uma análise multinível nos contextos individual, da assistência à saúde e socioeconômico*”, conducted within the standards of scientific ethics, approved by the Research Ethics Committee (Record in the CAAE – 35632414.5.0000.5190).

## RESULTS

Of the 9,001 deaths of children under one year registered in the Brazilian capitals in 2012, we could relate 90.8% of the DD to their respective DLB (Table 1).

**Table 1:** Absolute number and percentage of infant mortality recorded in the SIM paired with SINASC according to the type of linkage, capital, and average of the macro-regions. Brazil, 2012.

City	Total of deaths > 1 year	Paired deaths		Pairs – type of linkage			

				Deterministic		Probabilistic	
		
		n	%	n	%	n	%
Aracaju	144	138	95.8	137	95.1	1	0.7
Belém	366	295	80.6	208	56.8	87	23.8
Belo Horizonte	343	308	89.8	108	31.5	200	58.3
Boa Vista	90	77	85.6	50	55.6	27	30.0
Brasília	506	428	84.6	298	58.9	130	25.7
Campo Grande	117	115	98.3	115	98.3	0	0.0
Cuiabá	137	122	89.1	119	86.9	3	2.2
Curitiba	238	234	98.3	225	94.5	9	3.8
Florianópolis	50	47	94.0	38	76.0	9	18.0
Fortaleza	420	373	88.8	227	54.0	146	34.8
Goiânia	275	240	87.3	104	37.8	136	49.5
João Pessoa	159	152	95.6	126	79.2	26	16.4
Macapá	201	182	90.5	128	63.7	54	26.9
Maceió	229	209	91.3	83	36.2	126	55.0
Manaus	560	497	88.8	420	75.0	77	13.8
Natal	154	132	85.7	79	51.3	53	34.4
Palmas	44	40	90.9	13	29.5	27	61.4
Porto Alegre	180	177	98.3	177	98.3	0	0.0
Porto Velho	128	112	87.5	63	49.2	49	38.3
Recife	275	267	97.1	245	89.1	22	8.0
Rio Branco	79	73	92.4	0	0.0	73	92.4
Rio de Janeiro	1,120	1,039	92.8	730	65.2	309	27.6
Salvador	620	543	87.6	321	51.8	222	35.8
São Luís	275	239	86.9	176	64.0	63	22.9
São Paulo	2,024	1,897	93.7	1,897	93.7	0	0.0
Teresina	224	199	88.8	101	45.1	98	43.8
Vitória	43	42	97.7	38	88.4	4	9.3
Total	9,001	8,177	90.8	6,226	69.2	1,951	21.7

Source: SIM/SINASC – SVS/MS.

SIM: Mortality Information System; SINASC: Live Birth Information System

Porto Alegre, Curitiba, and Campo Grande presented the highest percentage of paired deaths. In Vitória and Recife, the proportion of associated records was expressive, exceeding 97%. On the other hand, the lowest percentages of linkage success were observed in Belém, Brasília, Boa Vista, and Natal. On average, the capitals of the South and Southeast regions presented better results in the linkage of the databases, while this result was lower in the capitals of the North region (Table 1).

Despite the variations in the linkage results between cities and regions, the success rate of database linkage was over 80% in all analysis units (Table 1).

In relation to the type of linkage, most pairs were obtained with the deterministic method (69.2%), while probabilistic linkage resulted in 21.7% of the concatenated records (Table 1).

In 22 capitals, we observed a greater contribution of the deterministic method for the pairing of the death and birth data. In cities such as Porto Alegre, Curitiba, Campo Grande, São Paulo, and Aracaju, the linkage results exceed 90% of pairing, and the first three cities reached more than 98% (Table 1).

On the other hand, in Rio Branco, Palmas, Belo Horizonte, Maceió, and Goiânia, paired records were predominant with the use of probabilistic linkage. We highlight Rio Branco, in which 92.4% of the deaths were related probabilistically. In Campo Grande, Porto Alegre, and São Paulo, this method had no contribution (Table 1).

In the average of all the regions, we observed a predominance of the deterministic linkage, which is more expressive in the South region. In the Northeast region, the probabilistic method contributed with 30.3% of the pairs, followed by the North region, with 26.8% (Table 1).

In the analysis of completeness of the 11 variables common to the analyzed systems, we found 11,278 unknown or unfilled fields in total, being 9,016 (79.9%) in the SIM and 2,262 (20.1%) in the SINASC. After linkage, we could recover 10,307 records, leaving only 971 incomplete fields, which is equivalent to a reduction of 91.4% in incompleteness. This increase in the completeness of the information was more significant for the SIM, which went from an average of 10% of incompleteness to 1.1%.

Moreover, in the total, regarding the categorization of the completeness of the variables studied before pairing in the SIM, we observed one excellent, six good, three regular, and one poor variable. For SINASC, eight of them were excellent and three were good. After linkage*,* all became excellent (Figure).

**Figure:**
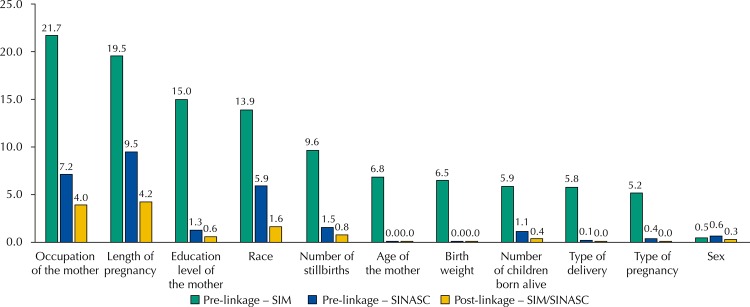
Percentage of incompleteness of the variables common to the SIM and SINASC, pre- and post-linkage of databases in the Brazilian capitals and Federal District. Brazil, 2012.

SIM: Mortality Information System; SINASC: Live Birth Information System

Among these variables, the sex of the child stands out, with excellent completeness in both systems. The occupation of the mother had the poorest quality, with 21.7% of the information being unknown in the SIM, followed by length of pregnancy, education level of the mother, and race. In the SINASC, length of pregnancy, occupation of the mother, and race also presented lower percentages of completeness. Nevertheless, the proportion of unfilled fields did not exceed 10% in any of them (Figure).

In the SIM, the variable of the sex of the child had excellent completion in all capitals even before linkage. The number of children born alive was also classified as excellent in 17 cities, among them Porto Alegre, Recife, Rio Branco, São Paulo, and Campo Grande, with less than 1% of unknown data (Table 2).

**Table 2:** Percentage of incompleteness in the SIM of the variables common to the SIM and SINASC, pre- and post-linkage of databases according to Brazilian capitals. Brazil, 2012.

City	Sex^a^	Race^b^	Age^c^	Edu. mother^d^	Occup. mother^e^	C. alive^f^	Stillbirth^g^	Type preg.^h^	Length preg.^i^	Delivery^j^	Weight^k^
										
	Pre | Post	Pre | Post	Pre | Post	Pre | Post	Pre | Post	Pre | Post	Pre | Post	Pre | Post	Pre | Post	Pre | Post	Pre | Post
Aracaju	1.4 | 1.4	31.2 | 0.0	0.0 | 0.0	0.0 | 0.0	10.1 | 4.3	3.6 | 0.0	7.2 | 0.0	0.0 | 0.0	2.9 | 0.0	0.0 | 0.0	0.0 | 0.0
Belém	0.1 | 0.7	0.7 | 0.0	5.4 | 0.0	10.5 | 0.3	6.4 | 0.0	5.4 | 0.3	7.8 | 0.3	3.1 | 0.0	20.7 | 2.0	5.8 | 0.0	6.8 | 0.0
Belo Horizonte	0.3 | 0.3	6.5 | 0.0	4.2 | 0.0	5.8 | 0.0	15.9 | 5.5	2.6 | 0.0	4.2 | 0.0	4.5 | 0.0	14.6 | 0.0	6.2 | 0.0	2.6 | 0.0
Boa Vista	0.0 | 0.0	18.2 | 0.0	6.5 | 0.0	15.6 | 0.0	23.4 | 0.0	11.7 | 0.0	26.0 | 0.0	9.1 | 0.0	29.9 | 0.0	9.1 | 0.0	13.0 | 0.0
Brasília	0.2 | 0.2	3.0 | 1.2	1.2 | 0.0	18.9 | 0.5	31.8 | 7.7	15.2 | 3.0	27.8 | 4.2	8.6 | 0.0	14.5 | 0.5	11.0 | 0.0	11.0 | 0.0
Campo Grande	0.0 | 0.0	0.0 | 0.0	0.9 | 0.0	1.7 | 0.0	0.9 | 0.0	0.9 | 0.0	0.9 | 0.0	0.0 | 0.0	1.7 | 0.0	0.0 | 0.0	0.9 | 0.0
Cuiabá	0.0 | 0.0	0.8 | 0.0	1.6 | 0.0	3.3 | 0.0	0.8 | 0.0	1.6 | 0.0	2.5 | 0.0	3.3 | 0.0	3.3 | 0.8	2.5 | 0.0	1.6 | 0.0
Curitiba	0.9 | 0.9	15.4 | 0.0	1.3 | 0.0	0.9 | 0.0	2.1 | 0.0	2.1 | 0.0	8.1 | 0.0	1.3 | 0.0	8.5 | 1.3	0.9 | 0.0	0.9 | 0.0
Florianópolis	0.0 | 0.0	2.1 | 0.0	4.3 | 0.0	10.6 | 0.0	6.4 | 0.0	10.6 | 0.0	14.9 | 0.0	2.1 | 0.0	10.6 | 4.3	2.1 | 0.0	2.1 | 0.0
Fortaleza	1.3 | 1.3	40.2 |11.5	10.2 | 0.0	29.8 | 1.9	36.5 | 2.1	7.2 | 0.0	11.3 | 0.0	9.1 | 0.3	23.3 | 5.4	8.6 | 0.0	8.3 | 0.0
Goiânia	0.8 | 0.8	18.8 | 4.6	15.8 | 0.0	26.7 | 1.3	59.6 | 6.7	4.2 | 0.0	3.8 | 0.0	10.8 | 0.0	17.1 | 1.7	12.9 | 0.0	12.1 | 0.0
João Pessoa	1.3 | 0.7	11.2 | 0.0	3.3 | 0.0	8.6 | 0.0	13.2 | 2.6	0.7 | 0.0	2.0 | 0.0	4.6 | 0.0	11.8 | 0.0	5.3 | 0.0	7.9 | 0.0
Macapá	0.0 | 0.0	19.8 | 2.7	13.2 | 0.0	31.9 | 1.1	47.8 | 16.5	23.6 | 0.0	35.7 | 0.0	13.7 | 0.0	39.6 | 4.9	14.8 | 0.0	14.8 | 0.0
Maceió	0.0 | 0.0	14.8 | 0.5	16.3 | 0.0	26.3 | 0.0	36.4 | 1.9	4.8 | 0.0	7.7 | 0.0	10.5 | 0.0	25.8 | 1.4	12.4 | 0.0	16.7 | 0.0
Manaus	0.8 | 0.4	10.9 | 0.2	6.2 | 0.0	14.5 | 0.2	12.1 | 1.6	3.8 | 0.0	6.2 | 0.0	5.2 | 0.0	13.7 | 3.4	5.2 | 0.0	6.6 | 0.2
Natal	0.0 | 0.0	22.0 | 0.0	27.3 | 0.0	35.6 | 1.5	56.8 | 19.7	3.8 | 0.0	6.8 | 0.0	21.2 | 0.0	32.6 | 2.3	22.0 | 0.0	25.0 | 0.0
Palmas	0.0 | 0.0	2.5 | 0.0	15.0 | 0.0	15.0 | 0.0	35.0 | 0.0	2.5 | 0.0	17.5 | 5.0	10.0 | 0.0	20.0 | 0.0	15.0 | 0.0	17.5 | 0.0
Porto Alegre	0.6 | 0.6	0.6 | 0.0	0.0 | 0.0	2.8 | 1.1	2.3 | 0.0	0.0 | 0.0	0.6 | 0.6	0.0 | 0.0	9.6 | 0.0	0.6 | 0.0	0.0 | 0.0
Porto Velho	0.9 | 0.0	16.1 | 1.8	25.9 | 0.0	57.1 | 2.7	52.7 | 6.3	17.9 | 0.0	20.5 | 0.0	15.2 | 0.0	34.8 | 9.8	13.4 | 0.0	14.3 | 0.0
Recife	0.4 | 0.4	5.2 | 0.0	0.7 | 0.0	1.1 | 0.4	2.2 | 0.0	0.0 | 0.0	4.9 | 0.0	0.4 | 0.0	2.2 | 0.0	0.7 | 0.0	0.4 | 0.0
Rio Branco	0.0 | 0.0	32.9 | 8.2	4.1 | 0.0	31.5 | 0.0	21.9 | 0.0	0.0 | 0.0	0.0 | 0.0	4.1 | 0.0	94.5 | 15.1	4.1 | 0.0	4.1 | 0.0
Rio de Janeiro	0.0 | 0.0	8.2 | 0.1	9.0 | 0.0	20.2 | 0.6	27.3 | 3.6	8.9 | 0.7	12.6 | 1.6	7.0 | 0.0	14.6 | 1.3	7.7 | 0.1	10.2 | 0.0
Salvador	1.8 | 0.7	34.3 | 1.7	14.7 | 0.0	33.7 | 0.2	69.2 | 11.8	18.6 | 2.0	26.5 | 4.2	11.2 | 0.2	20.4 | 5.7	12.9 | 0.0	13.4 | 0.0
São Luís	0.8 | 0.0	33.9 | 16.3	7.1 | 0.0	16.7 | 0.0	15.5 | 2.5	3.8 | 0.0	12.1 | 0.0	2.9 | 0.0	19.2 | 7.1	2.9 | 0.0	5.0 | 0.0
São Paulo	0.0 | 0.0	10.5 | 0.0	0.3 | 0.0	4.3 | 0.2	3.5 | 2.7	0.8 | 0.0	1.0 | 0.0	0.1 | 0.1	24.4 | 9.1	0.2 | 0.0	0.1 | 0.1
Teresina	0.0 | 0.0	16.1 | 0.3	11.1 | 0.0	16.6 | 1.5	33.2 | 3.5	5.0 | 0.0	13.6 | 0.0	5.0 | 0.0	38.7 | 10.6	5.5 | 0.0	8.5 | 0.0
Vitória	0.0 | 0.0	2.4 | 0.0	4.8 | 0.0	7.1 | 0.0	7.1 | 0.0	2.4 | 0.0	2.4 | 0.0	2.4 | 0.0	4.8 | 0.0	2.4 | 0.0	2.4 | 0.0

Source: SIM/SINASC – SVS/MS.

SIM: Mortality Information System; SINASC: Live Birth Information System

^a^ Sex of the child.

^b^ Race of the child.

^c^ Age of the mother.

^d^ Education level of the mother.

^e^ Occupation of the mother.

^f^ Number of children born alive.

^f^ Number of stillbirths.

^h^ Type of pregnancy.

^i^ Length of pregnancy.

^j^ Type of delivery.

^k^ Birth weight.

The variables of occupation of the mother, length of pregnancy, education level of the mother, and race were categorized as regular, poor, or very poor in more than half of the cities studied. The occupation of the mother was classified as very poor in four capitals – Salvador, Goiânia, Natal, and Porto Velho – and as poor in nine other ones. The length of pregnancy presented 94.5% of incompleteness in Rio Branco and between 20% and 40% in 11 capitals. The education level of the mother had 57.1% of missing data in Porto Velho, and it was identified as poor in eight other cities. The variable of race had poor completeness in six cities, among which Fortaleza stands out, with 40.2% (Table 2).

After the linkage of the databases, six variables became excellent in all cities in the SIM, namely: age and education level of the mother, number of children born alive, type of pregnancy, type of delivery, and birth weight. The variable of number of stillbirths was excellent in 26 capitals, except in Palmas. The variable of race in 24 cities and the occupation of the mother and length of pregnancy in 20 cities also became excellent. No variables were categorized as poor or very poor in the cities studied after linkage (Table 2).

Regarding the SINASC, five variables were considered as excellent before the linkage of the databases in all capitals: sex of the child, age of the mother, type of pregnancy, type of delivery, and birth weight. As excellent completeness, we can mention the education level of the mother and the number of children born alive in 25 cities and the number of stillbirths in 24 capitals (Table 3).

**Table 3:** Percentage of incompleteness in the SINASC of the variables common to the SIM and SINASC, pre- and post-linkage of databases according to Brazilian capitals. Brazil, 2012.

City	Sex^a^	Race^b^	Age^c^	Edu. mother^d^	Occup. mother^e^	C. alive^f^	Stillbirth^g^	Type preg.^h^	Length preg.^i^	Delivery^j^	Weight^k^
										
	Pre | Post	Pre | Post	Pre | Post	Pre | Post	Pre | Post	Pre | Post	Pre | Post	Pre | Post	Pre | Post	Pre | Post	Pre | Post
Aracaju	1.4 | 1.4	0.0 | 0.0	0.0 | 0.0	0.0 | 0.0	8.7 | 4.3	0.0 | 0.0	0.0 | 0.0	0.0 | 0.0	0.0 | 0.0	0.0 | 0.0	0.0 | 0.0
Belém	1.0 | 0.7	0.0 | 0.0	0.0 | 0.0	0.3 | 0.3	0.0 | 0.0	1.7 | 0.3	2.0 | 0.3	0.0 | 0.0	5.1 | 2.0	0.0 | 0.0	0.0 | 0.0
Belo Horizonte	0.3 | 0.3	1.0 | 0.0	0.0 | 0.0	0.3 | 0.0	24.0 | 5.5	0.0 | 0.0	0.0 | 0.0	0.0 | 0.0	1.3 | 0.0	0.0 | 0.0	0.0 | 0.0
Boa Vista	0.0 | 0.0	0.0 | 0.0	0.0 | 0.0	0.0 | 0.0	1.3 | 0.0	0.0 | 0.0	0.0 | 0.0	0.0 | 0.0	0.0 | 0.0	0.0 | 0.0	0.0 | 0.0
Brasília	0.7 | 0.2	24.5 | 1.2	0.0 | 0.0	3.5 | 0.5	18.5 | 7.7	5.8 | 3.0	7.2 | 4.2	0.5 | 0.0	6.1 | 0.5	0.5 | 0.0	0.0 | 0.0
Campo Grande	0.9 | 0.0	0.0 | 0.0	0.0 | 0.0	0.0 | 0.0	0.0 | 0.0	0.0 | 0.0	0.0 | 0.0	0.0 | 0.0	4.3 | 0.0	0.0 | 0.0	0.0 | 0.0
Cuiabá	0.0 | 0.0	1.6 | 0.0	0.0 | 0.0	0.0 | 0.0	0.0 | 0.0	0.0 | 0.0	0.0 | 0.0	0.0 | 0.0	2.5 | 0.8	0.0 | 0.0	0.0 | 0.0
Curitiba	0.9 | 0.9	0.4 | 0.0	0.0 | 0.0	0.0 | 0.0	0.0 | 0.0	0.0 | 0.0	0.0 | 0.0	0.4 | 0.0	17.9 | 1.3	0.0 | 0.0	0.0 | 0.0
Florianópolis	0.0 | 0.0	0.0 | 0.0	0.0 | 0.0	0.0 | 0.0	2.1 | 0.0	0.0 | 0.0	0.0 | 0.0	0.0 | 0.0	6.4 | 4.3	0.0 | 0.0	0.0 | 0.0
Fortaleza	1.3 | 1.3	26.0 | 11.5	0.0 | 0.0	2.9 | 1.9	3.8 | 2.1	0.0 | 0.0	0.0 | 0.0	0.5 | 0.3	17.7 | 5.4	0.0 | 0.0	0.0 | 0.0
Goiânia	1.7 | 0.8	18.8 | 4.6	0.0 | 0.0	2.1 | 1.3	12.1 | 6.7	0.0 | 0.0	0.0 | 0.0	0.8 | 0.0	15.0 | 1.7	0.8 | 0.0	0.0 | 0.0
João Pessoa	0.7 | 0.7	0.7 | 0.0	0.0 | 0.0	0.0 | 0.0	3.3 | 2.6	0.0 | 0.0	0.0 | 0.0	0.7 | 0.0	0.0 | 0.0	0.0 | 0.0	0.0 | 0.0
Macapá	1.1 | 0.0	3.8 | 2.7	0.0 | 0.0	1.1 | 1.1	18.1 | 16.5	0.0 | 0.0	0.0 | 0.0	0.0 | 0.0	9.9 | 4.9	0.0 | 0.0	0.0 | 0.0
Maceió	0.5 | 0.0	2.9 | 0.5	0.0 | 0.0	0.5 | 0.0	2.9 | 1.9	0.0 | 0.0	0.0 | 0.0	0.0 | 0.0	5.3 | 1.4	0.5 | 0.0	0.0 | 0.0
Manaus	0.4 | 0.4	2.0 | 0.2	0.0 | 0.0	0.8 | 0.2	4.8 | 1.6	0.0 | 0.0	0.0 | 0.0	0.0 | 0.0	14.9 | 3.4	0.0 | 0.0	0.2 | 0.2
Natal	0.0 | 0.0	0.8 | 0.0	0.0 | 0.0	5.3 | 1.5	22.0 | 19.7	0.0 | 0.0	0.0 | 0.0	0.0 | 0.0	8.3 | 2.3	0.0 | 0.0	0.0 | 0.0
Palmas	0.0 | 0.0	0.0 | 0.0	0.0 | 0.0	0.0 | 0.0	5.0 | 0.0	2.5 | 0.0	7.5 | 5.0	0.0 | 0.0	7.5 | 0.0	0.0 | 0.0	0.0 | 0.0
Porto Alegre	0.6 | 0.6	0.6 | 0.0	0.0 | 0.0	1.1 | 1.1	1.1 | 0.0	0.0 | 0.0	0.6 | 0.6	0.0 | 0.0	0.6 | 0.0	0.0 | 0.0	0.0 | 0.0
Porto Velho	0.0 | 0.0	7.1 | 1.8	0.0 | 0.0	3.6 | 2.7	7.1 | 6.3	0.0 | 0.0	0.0 | 0.0	0.9 | 0.0	22.3 | 9.8	0.9 | 0.0	0.0 | 0.0
Recife	1.1 | 0.4	0.0 | 0.0	0.0 | 0.0	0.4 | 0.4	0.0 | 0.0	0.0 | 0.0	0.0 | 0.0	0.0 | 0.0	0.7 | 0.0	0.0 | 0.0	0.0 | 0.0
Rio Branco	0.0 | 0.0	20.5 | 8.2	0.0 | 0.0	0.0 | 0.0	0.0 | 0.0	0.0 | 0.0	0.0 | 0.0	0.0 | 0.0	15.1 | 15.1	0.0 | 0.0	0.0 | 0.0
Rio de Janeiro	0.3 | 0.0	1.5 | 0.1	0.0 | 0.0	2.2 | 0.6	7.0 | 3.6	3.0 | 0.7	4.1 | 1.6	0.4 | 0.0	4.3 | 1.3	0.2 | 0.1	0.0 | 0.0
Salvador	0.9 | 0.7	5.7 | 1.7	0.0 | 0.0	2.4 | 2.0	14.5 | 11.8	5.5 | 2.0	7.4 | 4.2	1.7 | 0.2	14.0 | 5.7	0.4 | 0.0	0.0 | 0.0
São Luís	0.8 | 0.0	36.8 | 16.3	0.0 | 0.0	0.4 | 0.0	10.0 | 2.5	0.0 | 0.0	0.0 | 0.0	0.4 | 0.0	28.0 | 7.1	0.0 | 0.0	0.0 | 0.0
São Paulo	0.5 | 0.0	0.1 | 0.0	0.0 | 0.0	0.2 | 0.2	3.6 | 2.7	0.0 | 0.0	0.0 | 0.0	0.1 | 0.1	9.6 | 9.1	0.0 | 0.0	0.1 | 0.1
Teresina	0.0 | 0.0	22.1 | 3.0	0.0 | 0.0	5.0 | 1.5	9.5 | 3.5	0.0 | 0.0	0.0 | 0.0	3.5 | 0.0	24.1 | 10.6	1.0 | 0.0	0.0 | 0.0
Vitória	0.0 | 0.0	2.4 | 0.0	0.0 | 0.0	0.0 | 0.0	11.9 | 0.0	0.0 | 0.0	0.0 | 0.0	0.0 | 0.0	2.4 | 0.0	0.0 | 0.0	0.0 | 0.0

Source: SIM/SINASC – SVS/MS.

SIM: Mortality Information System; SINASC: Live Birth Information System

^a^ Sex of the child.

^b^ Race of the child.

^c^ Age of the mother.

^d^ Education level of the mother.

^e^ Occupation of the mother.

^f^ Number of children born alive.

^f^ Number of stillbirths.

^h^ Type of pregnancy.

^i^ Length of pregnancy.

^j^ Type of delivery.

^k^ Birth weight.

Race was the poorest variable in the highest number of cities, among which we can highlight São Luís – where incompleteness reached 36.8% –, Fortaleza, Brasília, Teresina, and Rio Branco. Length of pregnancy was categorized as poor in São Luís, Teresina, and Porto Velho, and as regular in six other cities. The occupation of the mother had poor completeness in Belo Horizonte and Natal, and regular in six cities (Table 3).

After linkage, seven variables were identified with excellent completeness in all cities in the SINASC, with the exception of race, occupation of the mother, number of stillbirths, and length of pregnancy. Race went from poor to regular in São Luís and Fortaleza. Another variable with regular status was the occupation of the mother, thus classified in Natal, Macapá, and Salvador. Length of pregnancy remained as 15.1% of incompleteness in Rio Branco and went to 10.6% in Teresina. At the end of the linkage of the databases, no variables were categorized as poor or very poor in the capitals (Table 3).

When analyzing the completeness of the variables according to capitals, two cities presented all the variables categorized as excellent for both the SIM and SINASC, even before linkage (Campo Grande and Cuiabá). In addition, four other capitals had 11 excellent variables in the SINASC before the linkage of the databases, which went to 14 in both systems after pairing (Tables 2 and 3).

Campo Grande, Cuiabá, Porto Alegre, Recife, Vitória, and São Paulo were the capitals with the best quality in completeness of information in the SIM, before linkage, with more than nine variables classified as excellent. In the SINASC, 22 cities presented more than 80% of excellent variables, and we highlight Campo Grande, Cuiabá, Porto Alegre, Recife, João Pessoa, and Boa Vista, as they presented all variables in this category (Tables 2 and 3).

Salvador, São Luís, Fortaleza, Rio Branco, Macapá, Natal, and Teresina, capitals of the North and Northeast regions, had the highest percentage of incompleteness of information, even after linkage (Tables 2 and 3).

We identified a significant association between the city of birth of the child, the component of infant mortality, and the success of the linkage between databases (Table 4).

**Table 4:** Absolute number, percentage, odds ratio (OR), and respective 95% confidence intervals of registered infant mortality, according to the pairing between the SIM and SINASC by Brazilian capital and average of macro-regions. Brazil, 2012.

Variable	Non-pair		Pair		OR	95%CI	χ^2^	p
	
	n	%	n	%				
North Region								
Belém	71	19.4	295	80.6	2.39	1.82–3.12	41.53	< 0.001
Boa Vista	13	14.4	77	85.6	1.67	0.93–3.03	2.38	0.061
Macapá	19	9.5	182	90.5	1.04	0.64–1.67	0.00	0.491
Manaus	63	11.3	497	88.8	1.26	0.96–1.65	2.51	0.056
Palmas	4	9.1	40	90.9	0.99	0.35–2.78	0.06	0.402
Porto Velho	16	12.5	112	87.5	1.42	0.83–2.40	1.31	0.097
Rio Branco	6	7.6	73	92.4	0.81	0.35–1.88	0.08	0.388
Total	192	13.1	1276	86.9	1.49	1.26–1.77	21.7	< 0.001
Northeast Region								
Aracaju	6	4.2	138	95.8	0.43	0.19–0.98	3.69	0.027
Fortaleza	47	11.2	373	88.8	1.25	0.91–1.71	1.78	0.093
João Pessoa	7	4.4	152	95.6	0.46	0.21–0.98	3.72	0.003
Maceió	20	8.7	209	91.3	0.95	0.59–1.51	0.10	0.459
Natal	22	14.3	132	85.7	1.65	1.05–2.61	4.16	0.021
Recife	8	2.9	267	97.1	0.30	0.15–0.60	11.9	0.002
Salvador	77	12.4	543	87.6	1.41	1.09–1.80	6.90	0.004
São Luís	36	13.1	239	86.9	1.50	1.04–2.14	4.46	0.017
Teresina	25	11.2	199	88.8	1.25	0.82–1.90	0.83	0.182
Total	248	9.9	2252	90.1	1.09	0.94–1.27	1.27	0.131
Southeast Region								
Belo Horizonte	35	10.2	308	89.8	1.13	0.79–1.61	0.32	0.286
Rio de Janeiro	81	7.2	1039	92.8	0.77	0.61–0.98	4.52	0.017
São Paulo	127	6.3	1897.	93.7	0.66	0.55–0.81	17.02	< 0.001
Vitória	1	2.3	42	97.7	0.24	0.03–1.72	1.65	0.099
Total	244	6.9	3286	93.1	0.74	0.63–0.85	16.1	< 0.001
South Region								
Curitiba	4	1.7	234	98.3	0.17	0.06–0.46	14.9	< 0.001
Florianópolis	3	6.0	47	94.0	0.63	0.197–2.04	0.28	0.299
Porto Alegre	3	1.7	177	98.3	0.17	0.05–0.53	11.18	< 0.001
Total	10	2.1	458	97.9	0.22	0.11–0.40	26.41	< 0.001
Midwest Region								
Brasília	78	15.4	428	84.6	1.81	1.41–2.33	21.14	< 0.001
Campo Grande	2	1.7	115	98.3	0.17	0.04–0.69	6.89	0.004
Cuiabá	15	10.9	122	89.1	1.22	0.71–2.10	0.33	0.283
Goiânia	35	12.7	240	87.3	1.45	1.01–2.08	3.64	0.028
Total	130	12.6	905	87.4	1.42	1.17–1.74	12.1	< 0.001
Grand Total	824	9.2	8177	90.8	1.00	-	-	-
Components								
Neonatal	395	6.4	5805	93.6	1.00	-	-	-
Post-Neonatal	429	15.3	2372	84.7	2.66	2.30–3.07	184.5	< 0.001
Total	824		8177					

Source: SIM/SINASC – SVS/MS.

SIM: Mortality Information System; SINASC: Live Birth Information System

Taking all capitals as reference, we can observe that this association presented statistical significance (p > 0.05) in 15 cities. In seven of them, there was a greater chance of non-pairing between the SIM and SINASC, among which we highlight Belém, Brasília, and Natal, with the highest values of OR. On the other hand, eight capitals had a lower chance of non-linkage of the significant records, with values of OR lower than 1.00, especially Campo Grande, Porto Alegre, Curitiba, and Recife (Table 4).

In the aggregate analysis of capitals by macro-region, the North and Midwest regions had greater probability of non-pairing. In the North region, four of the seven capitals had greater probability of non-linkage between databases. In the South and Southeast regions, we identified lower chances of linkage failure, with values of OR lower than 1.00 with statistical significance. This association was not significant only for the Northeast, being close to the average of the capitals (Table 4).

Regarding the death component, we found that, overall, deaths in the post-neonatal period had a greater chance of non-pairing of the death certificate records in relation to the respective DLB, when compared to neonatal deaths (Table 4).

## DISCUSSION

The high percentage of linkage success between the SIM and SINASC for the total Brazilian capital indicates the quality of the information of these systems. However, we observed regional differences, with better results in the capitals of the South and Southeast regions, while the pairing ratio was lower in the capitals of the North region. The proportion of paired records exceeded 97% in cities such as Porto Alegre, Curitiba, Campo Grande, Vitória, and Recife. The lowest percentages were found in Belém, Brasília, Boa Vista, and Natal, with values above 80%.

These results corroborate the findings of other studies^15–17^ and were superior when compared to previous research studies, whose linkage of birth and death databases resulted in percentages of pairs ranging from 40% to approximately 70%^20–22^.

We also highlight the predominance of the deterministic method to obtain paired records in 22 capitals. This result is related to the record of the number of the DLB, a univocal variable to the SIM and SINASC and of mandatory fulfillment for the deaths of children under one year. In cities such as Porto Alegre, Curitiba, and Campo Grande, the deterministic linkage results reached more than 98%. In contrast, we could not deterministically retrieve any records for Rio Branco, and the data was only correlated using the probabilistic method, as the city presents an expressive gap in the completeness of the DLB number in the SIM.

Similar results were observed in a study that has analyzed the contribution of linkage between the SIM and SINASC in five Brazilian cities, showing the predominance of the deterministic method^13^. Mendes et al.^14^ also discuss the relationship between the type of linkage and the quality of the information, verifying that, the larger the municipality, the more the records are paired deterministically and the smaller the occurrence of non-pairs; conversely, the smaller the municipality, the greater the contribution of the probabilistic method and the larger number of non-paired records.

The evaluation of completeness allows us to measure the frequency of information that is “unknown” or “unfilled”. Unknown variables are the product of a series of deficiencies, such as lack of information in the medical records and lack of knowledge of certain information by the woman’s companions, while unfilled variables are a reflection of the lack of care and importance given by the responsible professional to the filling of the information^22^.

Among the results of this study, regarding the analysis of completeness of the 11 variables common to the SIM and SINASC, we highlight the significant number of unknown or unfilled fields retrieved after linkage. For all 27 capitals, this contribution was greater for the SIM, which presented only one excellent variable before linkage, and all variables became excellent after the application of this technique.

Research studies that have used linkage between these information systems also suggest a more expressive contribution of SINASC to the SIM^15–17^. This is because, in general, the SINASC still presents superior quality to the death data registered in the SIM, both in terms of coverage and completeness and reliability of the information^3^ ^,^ ^7^ ^,^ ^8^ ^,^ ^14^.

The SINASC presented, even before linkage, good to excellent completion for most variables. A study that has analyzed the completeness of information in the DLB and the report of early neonatal and fetal death in the region of Ribeirão Preto, State of São Paulo, Brazil, has observed less than 10% of DLB with unfilled fields during the period from 2000 to 2007, and it also has detected an increasing trend in the quality of the filling^18^. These findings also resemble the research studies carried out in the Northeast region and in Pernambuco^11^ ^,^ ^23^.

In relation to the SIM, this study found high percentages of incompleteness. Only the variable of sex of the child was considered as excellent before linkage in the aggregate analysis of capitals. These deficiencies in the filling of important variables results in limitations in the potential use of the system for epidemiological studies^24^.

Among the variables studied, we highlight that the occupation of the mother, education level of the mother, race of the child, and length of pregnancy had lower filling quality, being categorized as regular, poor, or very poor in more than half of the Brazilian capitals, with a more significant percentage of incompleteness in the SIM. These findings corroborate what has been found in the literature^6^ ^,^ ^11^ ^,^ ^15^ ^,^ ^18^.

Maternal variables such as education level, occupation, and age, as well as race of the child, are considered important indicators of the socioeconomic conditions of the woman. In addition to problems related to the methodological clarity of the instructions for collecting and completing these fields, Romero and Cunha^10^ also suggest a correlation between completeness and the indicators of poverty, economic inequality, and human resources in health. In addition, the omission of data on these variables hinders studies on social disparities and infant mortality^6^.

We also highlight the high percentage of incompleteness of data on length of pregnancy in the SIM, which is an important predictor of infant mortality^19–21^. This variable was categorized as poor in 11 capitals studied, and we highlight Rio Branco, where the proportion of unfilled fields reached 94.5%. The application of linkage allowed us to retrieve information, and only two cities had regular completeness, while the other capitals reached the status of excellent or good.

The number of children born alive and the number of stillbirths, variables related to maternal parity, had good completeness in the SIM and excellent completeness in the SINASC for all capitals. These results differ from the research that has evaluated the quality of the information in the SINASC in the States of Brazil, showing that the parity variables were among those that showed greater incompleteness and lower consistency^12^.

Information related to the newborn, such as sex and birth weight, as well as the type of pregnancy and type of delivery, presented a very low frequency of unknown information for both the SIM and SINASC, corroborating the findings of other studies^11^ ^,^ ^12^ ^,^ ^15^ ^,^ ^22^.

Deaths in the post-neonatal period had a greater chance of non-pairing between the SIM and SINASC. This finding reinforces the precept that the investigation of infant mortality is more timely and robust the closer the birth and death events. In addition, we highlight the high occurrence of neonatal deaths in the hospital setting, which allows the better filling of information^13^ ^,^ ^14^.

We could also identify differences in the quality of information among the Brazilian capitals, as well as the completeness of the variables, as to the results of the linkage between databases, although with heterogeneous realities within the regions.

In the capitals of the North and Northeast regions of Brazil, such as Salvador, São Luís, Fortaleza, Rio Branco, Macapá, Natal, and Teresina, we found the highest percentage of incompleteness of information, even after pairing. In addition, the North and Midwest regions presented a lower chance of linkage success in relation to the other capitals, with statistical significance.

The quality of the records is related to the human and technological development conditions of each region ^24^, and it is considered a way of expressing inequities in the health care of more vulnerable groups, particularly the barriers to access to services^25^. It also reveals obstacles in the generation and consolidation of health information, such as: little importance given to the adequate filling of information by physicians and administrative staff, problems related to clarity in the instructions provided by the Ministry of Health, failures in typing the data in the system, and deficiencies in the corrections of the SIM database after the death investigation process^6^ ^,^ ^12^ ^,^ ^23^ ^,^ ^26^.

Despite advances in the coverage and quality of birth and death information systems in recent decades, the results of this study reinforce the existence of limitations. These problems were identified especially in the SIM and they are related to the filling of the death certificate and the completeness of the variables, which restrict the research on the risk factors for infant mortality.

This study indicated the linkage method between the SIM and SINASC for the qualification of databases, enabling the recovery of unfilled or unknown information of important variables for the analysis of infant mortality.

Given its low operational cost and ease of execution, we recommend the incorporation of linkage allied to the surveillance of infant mortality in the routine management of the SUS in its different spheres to improve information on health, understood as a strategic element for the analysis of the health situation and consequent decision making.

This study also suggests the need for investigations regarding other aspects of the quality of vital statistics, such as the presence of filling errors, identification of inconsistencies, and analysis of agreement between records of the same individual present in the different information systems.

## References

[1] Victora CG, Aquino EML, Leal MC, Monteiro CA, Barros FC, Szwarcwald CL (2011). Maternal and child health in Brazil: progress and challenges. Lancet.

[2] Barros FC, Matijasevich A, Requejo JH, Giugliani E, Maranhão AG, Monteiro CA (2010). Recent trends in maternal, newborn, and child health in Brazil: progress toward Millennium Development Goals 4 and 5. Am J Public Health.

[3] Mello Jorge MHP, Laurenti R, Gotlieb SLD (2007). Análise da qualidade das estatísticas vitais brasileiras: a experiência de implantação do SIM e do SINASC. Cienc Saude Coletiva.

[4] Frias PG, Pereira PMH, Andrade CLT, Szwarcwald CL (2008). Sistema de Informações sobre Mortalidade: estudo de caso em municípios com precariedade dos dados. Cad Saude Publica.

[5] Szwarcwald CL, Leal MC, Andrade CLT, Souza PRB (2002). Estimação da mortalidade infantil no Brasil: o que dizem as informações de óbitos e nascimentos do Ministério da Saúde?. Cad Saude Publica.

[6] Pedraza DF (2012). Qualidade do Sistema de Informações sobre Nascidos Vivos (SINASC): análise crítica da literatura. Cienc Saude Coletiva.

[7] Frias PG, Pereira PMH, Andrade CLT, Lira PIC, Szwarcwald CL (2010). Avaliação da adequação das informações de mortalidade e nascidos vivos no Estado de Pernambuco, Brasil. Cad Saude Publica.

[8] Frias PG, Szwarcwald CL, Lira PIC (2014). Avaliação dos sistemas de informações sobre nascidos vivos e óbitos no Brasil na década de 2000. Cad Saude Publica.

[9] Andrade CLT, Szwarcwald CL (2007). Desigualdades sócio-espaciais da adequação das informações de nascimentos e óbitos do Ministério da Saúde, Brasil, 2000-2002. Cad Saude Publica.

[10] Marques LJP, Oliveira CM, Bonfim CV (2016). Avaliação da completude e da concordância das variáveis dos Sistemas de Informações sobre Nascidos Vivos e sobre Mortalidade no Recife-PE, 2010-2012. Epidemiol Serv Saude.

[11] Silva RS, Oliveira CM, Ferreira DKS, Bonfim CV (2013). Avaliação da completitude das variáveis do Sistema de Informações sobre Nascidos Vivos – Sinasc – nos Estados da região Nordeste do Brasil, 2000 e 2009. Epidemiol Serv Saude.

[12] Romero DE, Cunha CB (2007). Avaliação da qualidade das variáveis epidemiológicas e demográficas do Sistema de Informações sobre Nascidos Vivos, 2002. Cad Saude Publica.

[13] Hofvendahl EA (1995). Smoking in pregnancy as a risk factor for long‐term mortality in the offspring. Paediatr Perinat Epidemiol.

[14] Mello Jorge MHP, Laurenti R, Gotlieb SLD (2010). Avaliação dos Sistemas de Informação em Saúde no Brasil. Cad Saude Coletiva.

[15] Maia LTS, Souza WV, Mendes ACG (2015). A contribuição do linkage entre o SIM e SINASC para a melhoria das informações da mortalidade infantil em cinco cidades brasileiras. Rev Bras Saude Mater Infant.

[16] Mendes ACG, Lima MM, Sá DA, Oliveira LCS, Maia LTS (2012). Uso da metodologia de relacionamento de bases de dados para qualificação da informação sobre mortalidade infantil nos municípios de Pernambuco. Rev Bras Saude Mater Infant.

[17] Marques LJP, Oliveira CM, Bonfim CV (2016). Avaliação da completude e da concordância das variáveis dos Sistemas de Informações sobre Nascidos Vivos e sobre Mortalidade no Recife-PE, 2010-2012. Epidemiol Serv Saude.

[18] Barbuscia DM, Rodrigues AL (2011). Completude da informação nas Declarações de Nascido Vivo e nas Declarações de Óbito, neonatal precoce e fetal, da região de Ribeirão Preto, São Paulo, Brasil, 2000-2007. Cad Saude Publica.

[19] Maia LTS, Souza WV, Mendes ACG (2012). Diferenciais nos fatores de risco para a mortalidade infantil em cinco cidades brasileiras: um estudo de caso-controle com base no SIM e no SINASC. Cad Saude Publica.

[20] Martins EF, Velásquez-Meléndez G (2004). Determinantes da mortalidade neonatal a partir de uma coorte de nascidos vivos, Montes Claros, Minas Gerais, 1997-1999. Rev Bras Saude Mater Infant.

[21] Nascimento EMR, Costa MCN, Mota ELA, Paim JS (2008). Estudo de fatores de risco para óbitos de menores de um ano mediante compartilhamento de bancos de dados. Cad Saude Publica.

[22] Costa JMBS, Frias PG (2009). Avaliação da completitude das variáveis da Declaração de Nascido Vivo de residentes em Pernambuco, Brasil, 1996 a 2005. Cad Saude Publica.

[23] Ramalho MOA, Frias PG, Vanderlei LCM, Macedo VC, Lira PIC (2015). Avaliação da incompletude de óbitos de menores de um ano em Pernambuco, Brasil, 1999-2011. Cienc Saude Coletiva.

[24] Silva LP, Moreira CMM, Amorim MHC, Castro DS, Zandonade E (2014). Avaliação da qualidade dos dados do Sistema de Informações sobre Nascidos Vivos e do Sistema de Informações sobre Mortalidade no período neonatal, Espírito Santo, Brasil, de 2007 a 2009. Cienc Saude Coletiva.

[25] Vanderlei LCM, Navarrete MLV (2013). Mortalidade infantil evitável e barreiras de acesso à atenção básica no Recife, Brasil. Rev Saude Publica.

[26] Haraki CAC, Gotlieb SLD, Laurenti R (2005). Confiabilidade do Sistema de Informações sobre Mortalidade em município do sul do Estado de São Paulo. Rev Bras Epidemiol.

